# Serum cystatin C predicts vancomycin trough levels better than serum creatinine in hospitalized patients: a cohort study

**DOI:** 10.1186/cc13899

**Published:** 2014-05-29

**Authors:** Erin N Frazee, Andrew D Rule, Sandra M Herrmann, Kianoush B Kashani, Nelson Leung, Abinash Virk, Nikolay Voskoboev, John C Lieske

**Affiliations:** 1 Hospital Pharmacy Services, Mayo Clinic, 200 First Street SW, Rochester MN 55905, USA; 2 Division of Nephrology and Hypertension, Mayo Clinic, 200 First Street SW, Rochester MN 55905, USA; 3 Division of Epidemiology, Mayo Clinic, 200 First Street SW, Rochester MN 55905, USA; 4 Division of Pulmonary and Critical Care Medicine, Mayo Clinic, 200 First Street SW, Rochester MN 55905, USA; 5 Division of Infectious Diseases, Mayo Clinic, 200 First Street SW, Rochester MN 55905, USA; 6 Department of Laboratory Medicine and Pathology, Mayo Clinic, 200 First Street SW, Rochester MN 55905, USA

## Abstract

**Introduction:**

Serum cystatin C can improve glomerular filtration rate (GFR) estimation over creatinine alone, but whether this translates into clinically relevant improvements in drug dosing is unclear.

**Methods:**

This prospective cohort study enrolled adults receiving scheduled intravenous vancomycin while hospitalized at the Mayo Clinic in 2012. Vancomycin dosing was based on weight, serum creatinine with the Cockcroft-Gault equation, and clinical judgment. Cystatin C was later assayed from the stored serum used for the creatinine-based dosing. Vancomycin trough prediction models were developed by using factors available at therapy initiation. Residuals from each model were used to predict the proportion of patients who would have achieved the target trough with the model compared with that observed with usual care.

**Results:**

Of 173 patients enrolled, only 35 (20%) had a trough vancomycin level within their target range (10 to 15 mg/L or 15 to 20 mg/L). Cystatin C-inclusive models better predicted vancomycin troughs than models based upon serum creatinine alone, although both were an improvement over usual care. The optimal model used estimated GFR by the Chronic Kidney Disease Epidemiology Collaborative (CKD-EPI) _creatinine-cystatin C_ equation (*R*^*2*^ = 0.580). This model is expected to yield 54% (95% confidence interval 45% to 61%) target trough attainment (*P* <0.001 compared with the 20% with usual care).

**Conclusions:**

Vancomycin dosing based on standard care with Cockcroft-Gault creatinine clearance yielded poor trough achievement. The developed dosing model with estimated GFR from CKD-EPI_creatinine-cystatin C_ could yield a 2.5-fold increase in target trough achievement compared with current clinical practice. Although this study is promising, prospective validation of this or similar cystatin C-inclusive dosing models is warranted.

## Introduction

The use of vancomycin, an anti-infective active against Gram-positive organisms, has increased nearly 100-fold over the last three decades [1-3]. This surge in utilization likely reflects the growing prevalence of staphylococcal infections, often methicillin-resistant *Staphylococcus aureus* (MRSA), for which vancomycin is considered first-line therapy [4,5]. Since vancomycin has a narrow therapeutic window, routine therapeutic drug monitoring with serum trough concentrations is recommended [4,5]. Unfortunately, recent evidence suggests that vancomycin dosing in clinical practice fails to reach trough targets in more than 50% of patients [6].

Although the precise reason for failure to achieve vancomycin targets is unknown, it may pertain to suboptimal assessment of glomerular filtration rate (GFR) because nearly 90% of the drug is eliminated renally [5,7,8]. The GFR in vancomycin dosing algorithms is most commonly estimated by Cockcroft-Gault creatinine clearance [8-10]. Unfortunately, these dosing algorithms were developed before serum creatinine assays were standardized. Also, creatinine-based GFR estimates are influenced by non-GFR factors not adequately accounted for by adjustments for age, sex, ethnicity, and weight. Finally, rapid changes in GFR among acutely ill patients are poorly captured by serum creatinine monitoring [11-19].

Cystatin C is another endogenous biomarker that, in combination with serum creatinine, improves GFR estimation relative to creatinine alone [12,20-24]. Unfortunately, the literature on cystatin C-guided medication dosing remains limited [25-31]. Pharmacokinetic analyses suggest that cystatin C may better predict vancomycin clearance than creatinine, yet the relationship of this biomarker with steady-state trough levels remains unclear [26-28]. Furthermore, it is unknown whether the combination of creatinine and cystatin C can be used to predict vancomycin troughs better than either GFR marker used in isolation. The purpose of this study was to determine the optimal model to predict vancomycin trough levels using serum creatinine or cystatin C or both. Results suggest that vancomycin dosing algorithms that employ cystatin C should be developed for prospective validation.

## Materials and methods

### Setting and participants

This prospective cohort study enrolled hospitalized adults at the Mayo Clinic in Rochester, Minnesota, who received intravenous vancomycin between March and October 2012 and had Minnesota research authorization [32]. The Mayo Clinic Institutional Review Board approved the protocol and waived the need for informed consent because the study was considered minimal risk. Other eligibility criteria included the measurement of creatinine upon vancomycin initiation (enrollment creatinine), availability of stored serum from this same sample for cystatin C measurement, and a steady-state vancomycin level. Patients were excluded if they developed stage 2 or stage 3 acute kidney injury (AKI) at baseline or prior to the vancomycin level, because changing renal function would prohibit the achievement of a steady state during vancomycin dosing [33]. In such cases, clinicians routinely administer a single dose of vancomycin and perform serial serum concentration monitoring to determine the appropriate time for a re-dose. Patients who received vancomycin at an inconsistent dose or interval were also excluded as were individuals with a body mass index of greater than 32 kg/m^2^ due to altered vancomycin pharmacokinetics in obesity [34].

Institutionally endorsed vancomycin dosing and monitoring recommendations were in place throughout the study [5,10,11]. Briefly, a vancomycin loading dose of 20 to 30 mg/kg and a maintenance dose of 15 to 20 mg/kg based on actual body weight were recommended for all patients. Dosing intervals were informed primarily by the Cockcroft-Gault creatinine clearance [10,11,35]. Regimens were tailored by the care team according to severity of infection and other determinants of renal function (that is, urine output), if available. Ultimately, the primary service, supported by a clinical pharmacist, both without cystatin C levels, established an individualized vancomycin regimen for each patient. In accordance with national guidelines, each regimen was designed to achieve a target vancomycin trough level, either between 10 and 15 mg/L or between 15 and 20 mg/L, appropriate for the suspected or documented source(s) of infection [4,5]. Specifically, individuals with bacteremia, endocarditis, osteomyelitis, meningitis, or pneumonia with a suspected association with *S. aureus* were assigned a goal trough range of 15 to 20 mg/L. In all other cases, the target trough range assigned was 10 to 15 mg/L. Vancomycin dosing interval reflects GFR and the anticipated drug half-life. Using guideline recommendations, trough levels were all drawn immediately before the fourth dose of vancomycin to approximate steady-state conditions (4 to 5 half-lives) [4,5].

### Measures

Patient demographics (age, gender, race, and ethnicity), comorbid conditions, severity of illness, and admission diagnosis were each noted. Other abstracted data included source of infection, vancomycin dose and interval, and routine laboratory data. Vancomycin level was analyzed by using the Syva Emit^®^ 2000 Vancomycin Assay (Siemens Healthcare Diagnostics, Inc., Newark, DE, USA). For the 12 (7%) individuals with undetectable vancomycin levels (<5.0 mg/L), serum levels were recorded as 2.5 mg/L. Creatinine measurement was performed by using the standardized—isotope dilution mass spectrometry (IDMS) traceable—Roche enzymatic creatinine assay (Roche, Basel, Switzerland). In patients receiving intravenous catecholamines, known to interfere with enzymatic assays, an IDMS traceable Roche rate-Jaffe creatinine assay was used instead (Roche Cobas Integra 400 Plus chemistry analyzer). Cystatin C was measured on stored serum used for the enrollment creatinine with a particle-enhanced turbidimetric assay (Gentian AS, Moss, Norway). This assay is traceable to the same international certified cystatin C reference material (ERM-DA471/IFCC) used to develop the cystatin C-based Chronic Kidney Disease Epidemiology Collaborative (CKD-EPI) equations [12,36].

### Data analysis

Continuous data were summarized by using mean ± standard deviation (SD) or median with interquartile range depending on distribution. The Wilcoxon signed-rank test was used to detect intra-individual differences in GFR estimates. Frequencies (percentages) were used to describe discrete data. The Pearson’s chi-square test or Fisher’s exact test analyzed independent binary outcomes between groups, whereas the McNemar’s test was used for paired data, including trough achievement between dosing approaches.

**Figure 1:**
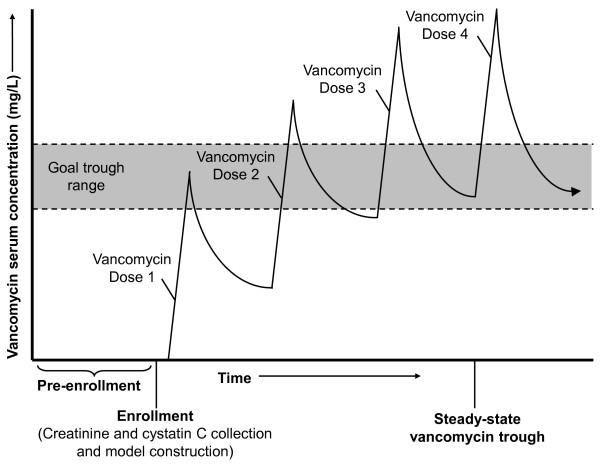
Patient enrollment flowchart.

Linear regression was used to develop predictive models for the vancomycin trough level (Figure 1). To develop models that would be clinically useful, predictors were included in the model only if readily available at baseline. Multivariate models included various combinations of age, sex, race, height (centimeters), weight (kilograms), body mass index (kilograms per square meter), baseline creatinine (milligrams per deciliter), and baseline cystatin C (milligrams per liter). Logarithmic transformation of serum creatinine and cystatin C improved model fit. The change in creatinine from pre-enrollment to enrollment (usually a 24-hour interval), total vancomycin dose received prior to trough (sum of the first three doses), use of a loading dose, dosing interval, and estimated GFR (eGFR) were also assessed. eGFR was determined by the Cockcroft-Gault equation and the three CKD-EPI equations (creatinine, cystatin C, or creatinine and cystatin C) re-expressed in milliliters per minute by multiplying by body surface area (BSA) derived from the Du Bois formula divided by 1.73 m^2^[12,37].

The target range for the vancomycin trough (10 to 15 mg/L or 15 to 20 mg/L) was based on the clinical indication for treatment. We analyzed our regression models to determine how well they would be expected to achieve vancomycin troughs within target ranges compared with usual clinical practice. First, model residuals for each patient were calculated on the basis of the differences between predicted and observed vancomycin trough levels. Model residuals represent all the unpredictable variation in vancomycin trough levels. Then model residuals were added to the target trough level (either 12.5 if target trough range was 10 to 15 mg/L or 17.5 if target trough range was 15 to 20 mg/L) to determine the expected trough. Thus, the expected trough is based on both the predictable variation (12.5 or 17.5 mg/L) and the unpredictable variation (residual) from the regression model. The proportion of individuals with an expected trough within their target trough range was then calculated.

To address potential over-fitting of models for trough level prediction, cross-validation was performed on the model with the best fit (highest *R*^*2*^). The final model was re-derived by using 90% of the sample (n = 156) and validated in the remaining 10% (n = 17). This cross-validation was repeated 10 times to determine the mean proportion of expected levels within the target trough range across the 10 replications. All analyses were performed with JMP version 9 statistical software (SAS Institute Inc., Cary, NC, USA).

## Results

**Figure 2:**
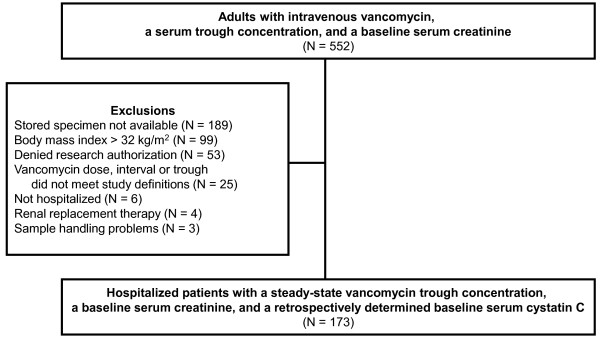
**Overview of the study protocol.** Factors available at baseline, prior to the first vancomycin dose, were used to develop vancomycin trough level prediction models. Steady-state vancomycin trough adequacy, prior to the fourth dose, was determined according to individualized target ranges based on suspected or documented source(s) of infection.

Of the 552 patients screened for eligibility, 173 individuals were enrolled in the study. The majority of excluded patients lacked an adequate stored specimen for cystatin C measurement (n = 189), were obese (n = 99), or declined research authorization for the study (n = 53) (Figure 2). No patients were excluded for KDIGO (Kidney Disease: Improving Global Outcomes) stage 2 or stage 3 AKI. The final cohort was 59 ± 16 (mean ± SD) years old, 54% male, and predominately Caucasian (95%). The mean BSA of included patients was 1.86 ± 0.2 m^2^ with a range of 1.29 to 2.52 m^2^. Moderate to severe chronic kidney disease with a baseline eGFR by CKD-EPI_creatinine-cystatin C_ of less than 60 mL/min was evident in 20% of patients (Table 1). The eGFR by CKD-EPI_cystatin C_ was significantly lower than by CKD-EPI_creatinine_ (−14 ± 25 mL/min, *P* <0.001). A creatinine value prior to the study baseline (Figure 1) was available in 122 (71%) individuals. Using this value, stage 1 AKI (an at least 0.3 mg/dL creatinine increase from baseline), though not an explicit exclusion criterion, affected only four (3%) patients in the included sample. The administered vancomycin maintenance dose compared well with institutional and national guidelines (16.3 ± 2.4 mg/kg). Dosing frequency differed from the institutional recommendations in 132 (76%) patients, most commonly because providers selected less frequent dosing intervals. The target troughs were 10 to 15 mg/L for 40% of patients and 15 to 20 mg/L for 60% of patients.

**Table 1:** Baseline patient characteristics, demographics, and infection characteristics

**Characteristic**	**Patients (n = 173)**^**a**^
Age, years	59 ± 16
Male, n (%)	93 (54)
Caucasian, n (%)	164 (95)
Body mass index, kg/m^2^	25.5 ± 3.6
Body surface area, m^2^	1.9 ± 0.2
Non-operative admission diagnosis, n (%)	110 (65)
Intensive care unit, n (%)	37 (22)
Renal parameters	
History of moderate to severe kidney disease, n (%)	22 (13)
Serum creatinine, mg/dL^b^	0.8 ± 0.4
Cystatin C, mg/L	1.1 ± 0.5
eGFR, mL/min^c^	
Cockcroft-Gault	108 ± 48
CKD-EPI_creatinine_	98 ± 29
CKD-EPI_cystatin C_	84 ± 37
CKD-EPI_creatinine-cystatin C_	91 ± 33
Infectious source, n (%)^d^	
Pulmonary	50 (29)
Bacteremia	36 (21)
Intra-abdominal	28 (16)
Skin and soft tissue infection	28 (16)
Musculoskeletal	27 (16)
Other/unknown^e^	43 (25)
Microbiology	
Culture positive, n (%)	98 (57)
Monomicrobial	51
Specific Gram-positive organisms isolated	
Coagulase-negative *Staphylococcus* spp.	25
Methicillin-susceptible *Staphylococcus aureus*	15
Methicillin-resistant *S. aureus*	10
*Enterococcus* spp.	22
Vancomycin	
Loading dose used, n (%)	64 (37)
Maintenance dose	
Non-weight based, mg	1,206 ± 263
Weight-based, mg/kg	16.3 ± 2.4
Dose between 14 and 21 mg/kg, n (%)	152 (88)
Interval, n (%)	
8 hours	8 (5)
12 hours	148 (86)
24 hours	17 (10)
Goal trough according to site of infection	
10-15 mg/L, n (%)	69 (40)
15-20 mg/L, n (%)	104 (60)
Trough level, mg/L	12.5 ± 6.0

^a^Values expressed as mean ± standard deviation unless noted. ^b^To convert the values for serum creatinine to micromoles per liter, multiply by 88.4. ^c^To convert from mL/min per 1.73^2^ multiply by [(0.007184*height (cm)^0.725^*weight (kg)^0.425^)/1.73] [37]. ^d^Sum of percentages exceeds 100 due to multiple sources of suspected/documented infection. ^e^Other sources include genitourinary, febrile neutropenia, cardiovascular, central nervous system, acute otitis media, and prophylaxis. CKD-EPI, Chronic Kidney Disease Epidemiology Collaborative; eGFR, estimated glomerular filtration rate; n, number.

**Figure 3:**
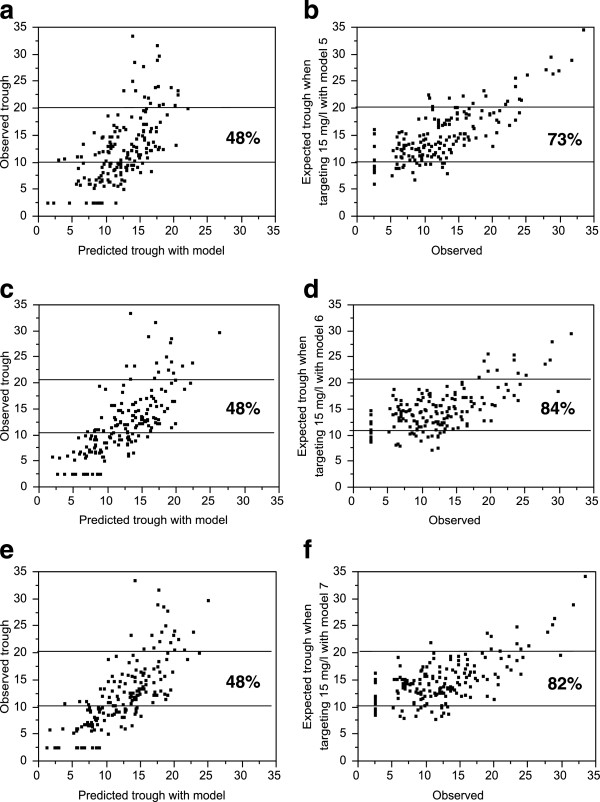
**Graphical representation of the expected improvement in vancomycin trough target levels with application of the Chronic Kidney Disease Epidemiology Collaborative models.** Predicted trough for each model (x-axis) versus actual trough or versus the expected trough when targeting 15 mg/L with the model (y-axis) for model 5 **(a,b)**, model 6 **(c,d)**, and model 7 **(e,f)**. The improvement in the proportion that would have an expected trough of 10 to 20 mg/L for each model is also shown. The more refined analysis targeting a trough of 10 to 15 or 15 to 20 mg/L (depending on the clinical indication for treatment) is presented in the text and tables.

Univariate analyses demonstrated a significant association between steady-state vancomycin trough level and each of the following variables: log creatinine, log cystatin C, eGFR, and total pre-trough vancomycin dose (Table 2). All multivariable models included vancomycin total dose and dosing interval. Separate models were developed by using the serum kidney function markers (serum creatinine or cystatin C or both) alone and as part of each of the four kidney function equations. Height was removed from the serum kidney function marker models as it was not a statistically significant predictor. Owing to the low prevalence of non-Caucasian race (5%), this predictor was also omitted. Models included age, sex, and weight, when not imbedded in GFR calculations. The models with the best fit and target trough achievement included cystatin C (Table 3 and Figure 3). The published CKD-EPI equations report eGFR in milliliters per minute per 1.73 m^2^[12]. Either converting CKD-EPI eGFR to milliliters per minute or adding weight improved model fit, but doing both did not further improve fit (Additional file 1).

**Table 2:** Univariate predictors of vancomycin trough level, mg/L

**Potential baseline predictor**	**Beta**	***P *** **value**
Demographic and anthropometric data		
Age, year	0.057	0.06
Male	−0.076	0.97
Height, cm	−0.015	0.72
Weight, kg	0.039	0.12
Kidney function markers		
Log creatinine, mg/dL	5.320	<0.001
Log cystatin C, mg/L	9.592	<0.001
Equations for estimated GFR, mL/min^a^		
Cockcroft-Gault	−0.041	<0.001
CKD-EPI_creatinine_	−0.076	<0.001
CKD-EPI_cystatin C_	−0.094	<0.001
CKD-EPI_creatinine-cystatin C_	−0.101	<0.001
Vancomycin parameters		
Loading dose given	1.412	0.15
Total pre-trough dose, g	1.635	0.004
Interval		
Every 8 hours	2.216	0.33
Every 12 hours	REF	-
Every 24 hours	−0.943	0.56

^a^To convert from mL/min per 1.73^2^ multiply by [(0.007184*height (cm)^0.725^*weight (kg)^0.425^)/1.73] [37]. CKD-EPI, Chronic Kidney Disease Epidemiology Collaborative; GFR, glomerular filtration rate; REF, reference.

**Table 3:** Predictive models for vancomycin trough level, mg/L

**Model variable **^**a**^	**Beta**	***P *** **value**	**Model fit (*R***^***2***^**)**	**Target trough achievement (95% CI)**
**Model 1: log creatinine**			0.306	35% (28%-42%)
Intercept	9.10	0.002		
Age, years	0.0886	0.002		
Male	−2.30	0.02		
Weight, kg	−0.0136	0.004		
Vancomycin total dose, g	3.28	<0.001		
Every 8-hour interval	3.18	0.1		
Every 12-hour interval	REF	-		
Every 24-hour interval	−6.61	<0.001		
Log baseline creatinine	8.80	<0.001		
**Model 2: log cystatin C**			0.559	52% (45%-59%)
Intercept	10.2	<0.001		
Age, years	−0.00578	0.8		
Male	−2.32	0.004		
Weight, kg	−0.0708	0.05		
Vancomycin total dose, g	2.50	<0.001		
Every 8-hour interval	4.25	0.009		
Every 12-hour interval	REF	-		
Every 24-hour interval	−6.66	<0.001		
Log baseline cystatin C	12.6	<0.001		
**Model 3: log creatinine and log cystatin C**			0.575	53% (45%-60%)
Intercept	11.5	<0.001		
Age, years	0.00582	0.8		
Male	−2.64	0.001		
Weight, kg	−0.0931	0.01		
Vancomycin total dose, g	2.70	<0.001		
Every 8-hour interval	4.15	0.009		
Every 12-hour interval	REF	-		
Every 24-hour interval	−7.90	<0.001		
Log baseline creatinine	2.89	0.01		
Log baseline cystatin C	11.3	<0.001		
**Model 4: eGFR with Cockcroft-Gault**			0.269	33% (26% - 40%)
Intercept	10.3	<0.001		
Vancomycin total dose, g	2.66	<0.001		
Every 8-hour interval	3.20	0.1		
Every 12-hour interval	REF	-		
Every 24-hour interval	−4.11	0.008		
eGFR with Cockcroft-Gault, mL/min	−0.0704	<0.001		
**Model 5: eGFR with CKD-EPI**_**creatinine**_			0.394	38% (31%-46%)
Intercept	16.4	<0.001		
Vancomycin total dose, g	3.35	<0.001		
Every 8-hour interval	3.47	0.06		
Every 12-hour interval	REF	-		
Every 24-hour interval	−7.58	<0.001		
eGFR with CKD-EPI_creatinine_, mL/min^b^	−0.163	<0.001		
**Model 6: eGFR with CKD-EPI**_**cystatin C**_			0.538	51% (44%-59%)
Intercept	14.3	<0.001		
Vancomycin total dose, g	2.61	<0.001		
Every 8-hour interval	4.91	<0.001		
Every 12-hour interval	REF	-		
Every 24-hour interval	−5.94	<0.001		
eGFR with CKD-EPI_cystatin C_, mL/min^b^	−0.134	<0.001		
**Model 7: eGFR with CKD-EPI**_**creatinine-cystatin C**_			0.580	54% (45%-61%)
Intercept	16.7	<0.001		
Vancomycin total dose, g	2.95	<0.001		
Every 8-hour interval	4.84	0.002		
Every 12-hour interval	REF	-		
Every 24-hour interval	−7.70	<0.001		
eGFR with CKD-EPI_creatinine-cystatin C_, mL/min^b^	−0.163	<0.001		

^a^Vancomycin total dose represents the cumulative grams of vancomycin given prior to trough level being drawn. Per the study definition, this represents three doses of vancomycin therapy. ^b^To convert from milliliters per minute per 1.73^2^, multiply by [(0.007184*height (cm)^0.725^*weight (kg)^0.425^)/1.73] [37]. CI, confidence interval; CKD-EPI, Chronic Kidney Disease Epidemiology Collaborative; eGFR, estimated glomerular filtration rate; REF, reference.

**Figure 4:**
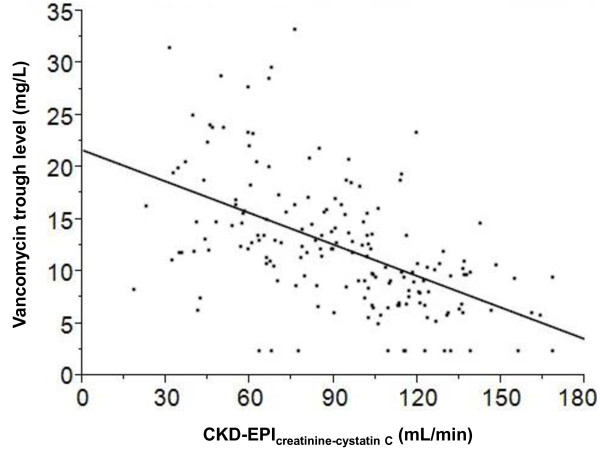
**Association between vancomycin trough concentrations and estimated glomerular filtration rate (GFR) with CKD-EPI**_**creatinine-cystatin C**_**.** A significant inverse relationship exists between GFR and vancomycin troughs (*P* <0.0001). Patients with a GFR of more than 120 mL/min rarely reached the minimum acceptable trough of 10 mg/L. CKD-EPI, Chronic Kidney Disease Epidemiology Collaborative.

The vancomycin trough achieved target in only 35 patients (20%) during routine clinical care with maintenance doses of 15 to 20 mg/kg and intervals informed by the Cockcroft-Gault creatinine clearance. Trough achievement in clinical practice was 22% when institutional dosing interval recommendations were followed compared with 20% when not followed (*P* = 0.8). Application of the model with the greatest power (model 7: eGFR with CKD-EPI_creatinine-cystatin C_; *R*^2^ = 0.580) is expected to yield about a 2.5-fold greater trough achievement than that which resulted from routine clinical practice (54% versus 20%; *P* <0.0001 versus observed trough). Among patients with CKD (defined as CKD-EPI_creatinine-cystatin C_ of less than 60 mL/min), the improvement between observed and expected trough achievement was modest (34% versus 43%, *P* = 0.4). In contrast, among patients without CKD (defined as CKD-EPI_creatinine-cystatin C_ of more than 60 mL/min), the improvement between observed and expected trough achievement was much greater (17% versus 57%, *P* <0.001). Notably, low trough levels were associated with a high GFR (Figure 4; *P* <0.0001). Cross-validation of model 7 to address potential overfitting of the data projected a slightly lower target trough achievement of 51% across the 10 replications instead of 54%.

## Discussion

In this study, only 20% of patients achieved a serum vancomycin trough level within their target range under usual care based on a combination of the Cockcroft-Gault equation, manufacturer guidelines, and clinician judgment. To determine whether clinically available data could be used to predict trough levels, we developed novel models based on serum creatinine, cystatin C, or eGFR with the Cockcroft-Gault and CKD-EPI equations. The optimal model used the CKD-EPI_creatinine-cystatin C_ equation for eGFR and is projected to improve trough achievement to greater than 50%. When these models were used, a practical set of dosing recommendations for vancomycin using the CKD-EPI equations could be developed for prospective testing.

Cystatin C, an endogenous proteinase inhibitor, is one of the first surrogates of GFR to emerge as a viable and practical alternative to creatinine in the last several decades [33,38]. Minimal data regarding the use of cystatin C-based medication dosing have been published. The present study demonstrated that the use of serum cystatin C, alone or with creatinine, improved expected target vancomycin trough achievement. Published pharmacokinetic analyses also suggest that cystatin C-based GFR estimates more accurately predict vancomycin clearance than creatinine clearance [25,29,30,39]. Furthermore, the current literature suggests that steady-state vancomycin troughs correlate more significantly with cystatin C-based models than creatinine-based models, largely due to reduced error among older individuals [26-28]. The present study demonstrates the potential utility of cystatin C to achieve desired vancomycin trough levels in a real-world setting.

Although there is much emphasis on drug dose adjustment for patients with CKD (GFR <60 mL/min), we found that the greatest improvement in target trough attainment was among patients without CKD (GFR >60 mL/min). Indeed, the cystatin C-based models particularly improved predicted troughs among those with a GFR of more than 120 mL/min, a group in whom underdosing frequently occurs under usual clinical care [40]. These findings are similar to those of another recent report which demonstrated that cystatin C performed better than creatinine clearance for vancomycin dosing when the serum creatinine was not more than 1.2 mg/dL [39].

Our study expands upon previous work because it accounts for other clinically relevant non-GFR factors in the predictive model, including the use of a vancomycin loading dose and the total dose given prior to the trough level. As expected, loading doses were not associated with predicted vancomycin trough since troughs were measured under steady-state conditions. Although vancomycin loading has the theoretical pharmacokinetic benefit of expediting the time to a higher serum drug concentration and potential clinical effect (not measured in this study), our study detected no impact of loading on steady-state blood levels among the 37% of patients in whom one was used [41]. Drug dosing recommendations by the US Food and Drug Administration and those used in clinical practice are based largely on GFR estimates expressed in milliliters per minute not corrected for BSA [26-28]. Therefore, also unlike most previous investigators, we converted eGFR to milliliters per minute using the Du Bois formula for BSA in order to remove the 1.73 m^2^ normalization factor [12,37]. The current models could be used to develop dosing algorithms that include eGFR, desired interval, and trough goal. If serum cystatin C levels are not available at therapy initiation, we suggest that initial recommendations could use the eGFR_creatinine_ model (model 5) with subsequent dose modification based on the eGFR_creatinine-cystatin C_ model (model 7) once available.

The exact reason why serum cystatin C performed better than serum creatinine for vancomycin trough prediction is not entirely clear. Numerous studies among stable ambulatory patients have shown that cystatin C-based equations perform similar to creatinine-based equations in estimating GFR when either marker is used in isolation [12,20,21]. However, cystatin C may more closely capture subtle fluctuations in GFR among acutely ill, hospitalized patients than serum creatinine [42]. Inflammation is an important non-GFR determinant of increased cystatin C levels [43,44]. In the hospital setting, rises in cystatin C could reflect inflammation secondary to infection, and the severity of infection, hence an increase in cystatin C, may predict a GFR decline which ultimately impacts vancomycin trough levels. The 15 mL/min lower GFR estimate by CKD-EPI_cystatin C_ than by CKD-EPI_creatinine_ is consistent with both hypotheses. In addition, the biggest improvement in troughs was among individuals with higher levels of GFR, a group in whom cystatin C appears to perform better than creatinine alone as a biomarker of GFR [12]. We also found that GFR estimation may not actually be a necessary step for a vancomycin dosing model. Models 2 and 3 use cystatin C without estimating GFR and perform similar to models 6 and 7. Furthermore, models 2 and 3 do not rely on the documentation of height. Given the historical reliance on GFR estimating equations for drug dosing, models using eGFR may be easier to implement in clinical practice.

There are several potential limitations of this study. The small proportion of non-Caucasian individuals included does not allow study of the impact that race and ethnicity could have on model performance. However, cystatin C GFR estimates have not been found to be significantly impacted by race, and the performance of the CKD-EPI equations in Caucasian populations has been established [12]. In addition, few patients in the present study were experiencing significant changes in renal function based upon serum creatinine at the time of inclusion. However, patients clearly developing AKI would not be dosed on a consistent schedule but instead would be dosed individually based upon random vancomycin levels. Indeed, any dosing algorithm will need to be suspended if the patient develops AKI. Patients with a body mass index of more than 32 kg/m^2^ were also excluded. Obesity alters the pharmacokinetics of vancomycin and affects cystatin C levels independent of GFR, and thus the performance of cystatin C-based dosing models in this population needs further study [34,43]. We emphasize that prospective testing of these models is needed to determine whether the expected improvement can be translated into actual improvement in target trough achievement. Also, though not the subject of the present study, future research should also evaluate whether any change in actual vancomycin target attainment with cystatin C-based dosing algorithms translates into improvements in therapeutic efficacy and safety.

## Conclusions

We found that a model based on eGFR from CKD-EPI_creatinine-cystatin C_ optimally predicts vancomycin trough levels. Target trough achievement (10 to 15 mg/L or 15 to 20 mg/L) is expected to be about 2.5-fold better with this model than current clinical practice. These findings are promising and encourage further investigation to prospectively validate the proposed or similar cystatin C-based vancomycin dosing models.

## Key messages

• Vancomycin dosing in hospitalized adults infrequently achieves recommended target trough levels, perhaps due to suboptimal assessment of glomerular filtration rate.

• A novel cystatin C-based dosing model which includes eGFR with the CKD-EPI_creatinine-cystatin C_ equation was developed and is projected to result in a 2.5-fold improvement in target trough attainment over usual care.

• Cystatin C is a promising GFR surrogate and further study is needed to evaluate its potential for medication dosing.

## Abbreviations

AKI: acute kidney injury; BSA: body surface area; CKD: chronic kidney disease; CKD-EPI: Chronic Kidney Disease Epidemiology Collaborative; eGFR: estimated glomerular filtration rate; GFR: glomerular filtration rate; IDMS: isotope dilution mass spectrometry; SD: standard deviation.

## Competing interests

The authors declare that they have no competing interests.

## Authors’ contributions

EF had full access to all of the data in the study and takes responsibility for the integrity of the data and the accuracy of the analysis. She helped to design the study, to gather data on included subjects in conjunction with the representatives of Mayo Validation Support Services cited in the Acknowledgments, to perform the statistical analysis, and to draft the manuscript. AR had full access to all of the data in the study and takes responsibility for the integrity of the data and accuracy of the analysis. He helped to design the study, to perform the statistical analysis, and to draft the manuscript. JL had full access to all of the data in the study and takes responsibility for the integrity of the data and accuracy of the analysis. He helped to design the study, to perform the statistical analysis, and to draft the manuscript. AV helped to design the study and to review the statistical analysis. SH and NV helped to gather data on included subjects in conjunction with the representatives of Mayo Validation Support Services cited in the Acknowledgments. KK and NL helped to review the statistical analysis. All authors reviewed the data, participated in discussions related to interpretation and read and approved the final manuscript.

## Supplementary Material

Additional file 1 **Chronic Kidney Disease Epidemiology Collaborative (CKD-EPI) estimated glomerular filtration rate (eGFR) predictive models for vancomycin trough with and without both body surface area normalization for 1.73 m**^**2 **^**and weight.** Click here for file
