# Supplementary material for: Serum cystatin C predicts vancomycin trough levels better than serum creatinine in hospitalized patients: a cohort study
Source: Crit Care. 2014 May 29;18(3):R110. doi: 10.1186/cc13899 (PMC4075252; doi:10.1186/cc13899)
Supplement: Additional file 1 — Chronic Kidney Disease Epidemiology Collaborative (CKD-EPI) estimated glomerular filtration rate (eGFR) predictive models for vancomycin trough with and without both body surface area normalization for 1.73 m 2 and weight. [file cc13899-S1.pdf]

Additional file 1. CKD-EPI eGFR predictive models for vancomycin trough with and without both BSA normalization for 1.73 m^2 and weight

Model variable [a]	Model fit (R^2)
**Model 5: eGFR with CKD $EPI_{creatinine}$**	
Intercept	
Vancomycin total dose (g)	
Every 8 hour interval	
Every 12 hour interval	
Every 24 hour interval	
**and**	
eGFR in mL/min/1.73m^2	0.330
eGFR in mL/min/1.73m^2 and weight	0.384
eGFR in mL/min [b]	0.394
eGFR in mL/min and weight [b]	0.396
**Model 6: eGFR with CKD $EPI_{cystatin\ C}$**	
Intercept	
Vancomycin total dose (g)	
Every 8 hour interval	
Every 12 hour interval	
Every 24 hour interval	
**and**	
eGFR in mL/min/1.73m^2	0.525
eGFR in mL/min/1.73m^2 and weight	0.544
eGFR in mL/min [b]	0.538
eGFR in mL/min and weight [b]	0.538
**Model 7: eGFR with CKD $EPI_{creatinine\text{-}cystatin\ C}$**	
Intercept	
Vancomycin total dose (g)	
Every 8 hour interval	
Every 12 hour interval	
Every 24 hour interval	
**and**	
eGFR in mL/min/1.73m^2	0.536
eGFR in mL/min/1.73m^2 and weight	0.574
eGFR in mL/min [b]	0.580
eGFR in mL/min and weight [b]	0.580

[a]. Vancomycin total dose represents the cumulative grams of vancomycin given prior to trough level being drawn. Per the study definition this represents three doses of vancomycin therapy.
[b] : To convert from mL/min/1.73^2 multiply by [(0.007184*Height $(cm)^{0.725}$*Weight $(kg)^{0.425}$)/1.73] [37]
